# Hepatoprotective effects of blue honeysuckle on CCl_4_‐induced acute liver damaged mice

**DOI:** 10.1002/fsn3.893

**Published:** 2018-11-27

**Authors:** You‐Suk Lee, Il Je Cho, Joo Wan Kim, Min‐Ki Lee, Sae Kwang Ku, Jae‐Suk Choi, Hae‐Jeung Lee

**Affiliations:** ^1^ Department of Food and Nutrition College of BioNano Technology Gachon University Seongnam‐si Gyeonggi‐do Korea; ^2^ The Medical Research Center for Globalization of Herbal Formulation Department of Herbal Formulation College of Oriental Medicine Daegu Haany University Gyeongsan‐si Gyeongdanuk‐do Korea; ^3^ Aribio Co. Ltd. Seongnam‐si Gyeonggi‐do Korea; ^4^ Department of Physical Education Kongju National University Kongju‐si Chngcheongnam‐do Korea; ^5^ Department of Anatomy and Histology College of Korean Medicine Daegu Haany University Gyeongsan‐si Gyeongdanuk‐do Korea; ^6^ Division of Bioindustry College of Medical and Life Sciences Silla University Busan Korea

**Keywords:** antioxidant, CCl_4_, liver, *Lonicera caerulea*, mice

## Abstract

The objective of this study was to evaluate the hepatoprotective effects of blue honeysuckle (BH) on carbon tetrachloride (CCl_4_)‐induced acute hepatic damage in mice. The experiment used a total of 60 ICR mice, which were divided into six groups. Except for the intact control groups, all groups received a single intraperitoneal injection of CCl_4_ after a 7 day pre‐treatment period with distilled water, BH extracts, or silymarin. Twenty‐four hours after the CCl_4_ injection, the following observations, representative of classical oxidative stress‐mediated centrolobular necrotic acute liver injuries, were observed: decreased body weight; small nodule formation and enlargement on the gross inspections with related liver weight increase; elevation of serum AST and ALT, increases in hepatic lipid peroxidation and related depletion of endogenous antioxidants and antioxidative enzymes; centrolobular necrosis; increases in apoptotic markers, lipid peroxidation markers, and oxidative stress markers. However, liver damage was significantly inhibited by the pre‐treatment with BH extracts. The present study demonstrated that oral administration of BH extracts prior to exposure to CCl_4_ conferred favorable hepatoprotective effects. These results demonstrated that BHe possessed suitable properties for use as a potent hepatoprotective medicinal food.

## 1 INTRODUCTION

The liver performs various pivotal functions, including protein synthesis, glucose homeostasis, detoxification, and the utilization of various nutrients (Lu et al., 2016; Yang, Zhang, Guan, & Hua, 2015). Generally, when the liver is exposed to high levels of environmental toxins, metabolic dysfunction of the liver may occur, which ranges from the transient elevation of liver enzymes to life‐threatening hepatic fibrosis, liver cirrhosis, and even hepatocellular carcinoma (Sun et al., 2011). Substantial evidence implicates oxidative stress and inflammation in the etiology of liver injury (Berasain et al., 2009). Similar effects are caused by CCl_4_, an industrial solvent known to induce liver injury and liver diseases, which is widely used in experimental hepatopathy (Yang et al., 2015; Zou, Qi, Ye, & Yao, 2016). CCl_4_‐induced toxicity depends on the dose and duration of exposure. At a low dose, transient effects occur, including the loss of Ca^2+^ sequestration, impaired lipid homeostasis, and the release of several cytokines. Longer exposures alter fatty acid metabolism and induce fibrosis, cirrhosis, and cancer (Cui, Yang, Lu, Chen, & Zhao, 2014). CCl_4_‐induced hepatotoxicity is the result of reductive dehalogenation reactions catalyzed by the hepatic cytochrome P‐450, which forms unstable trichloromethyl and trichloromethyl peroxyl radicals capable of binding to proteins or lipids and initiating lipid peroxidation and liver damage (Cheng et al., 2013). Oxidative stress has been accepted as one of the principal causes of CCl_4_‐induced hepatic injury, which is mediated by the production of free radical derivatives of CCl_4_ and is responsible for cell membrane damage and the subsequent release of the marker enzymes of hepatotoxicity (Boll, Weber, Becker, & Stampfl, 2001; Weber, Boll, & Stampfl, 2003). Inflammation is another important pathological mechanism through which CCl_4_‐induced liver injury is propagated (Ebaid, Bashandy, Alhazza, Rady, & El‐Shehry, 2013; Yang et al., 2015).

Therefore, the hepatoprotective effects of test materials are evaluated on CCl_4_‐induced acute liver damage through histopathological analyses and the examination of anti‐inflammatory potential and antioxidative activities (Ferreira et al., 2010; Wang et al., 2013).

Although the need for medicines to protect against liver damage has emerged, modern medicine still lacks reliable hepatoprotective drugs; therefore, numerous traditional herbal medicines have been studied for the evaluation of their hepatoprotective efficiency (Lu et al., 2016). Silymarin is a flavonoid found in the herb milk thistle, *Silybum marianum*. Milk thistle grows wild in a variety of settings, including roadsides. Silymarin is a powerful antioxidant that protects liver cells from toxins (Wellington & Jarvis, 2001). The antioxidant effects of silymarin on CCl_4_‐induced liver damage have been well documented (Cordero‐Pérez et al., 2013; Vargas‐Mendoza et al., 2014); therefore, it was selected as a reference agent in this study.

Blue honeysuckle (BH) is a shrub traditionally used in folk medicine in northern Russia, China, and Japan, but its fruits are also considered an edible berry in North America, Europe, and Korea (Svarcova, Heinrich, & Valentova, 2007). The berry is a rich source of ascorbic acid and phenolic components, particularly anthocyanins, flavonoids, and low molecular weight phenolic acids (Chaovanalikit, Thompson, & Wrolstad, 2004; Svarcova et al., 2007). These compounds have been reported to exert multiple biological effects, including strong antioxidant activity (Svarcova et al., 2007). Recently, it was reported that orally administered BH protected mice against ionizing radiation (Zhao et al., 2012), ameliorated abnormal lipid and glucose metabolism in rats (Jurgoński, Juśkiewicz, & Zduńczyk, 2013), and exhibited hepatoprotective (Palíková, Valentová, Oborná, & Ulrichová, 2009), anti‐inflammatory (Jin et al., 2006; Zdařilová, Svobodova, Chytilová, Šimánek, & Ulrichová, 2010), and therapeutic effects on hyperthyroidism (Park et al., 2016). In particular, BH extracts showed the strongest antioxidant potential among 12 different colored berries (Chen, Xin, Yuan, Su, & Liu, 2014); the phenolic‐rich extract of BH has been shown to possess anti‐inflammatory and wound‐healing effects in vitro and in vivo (Jin et al., 2006) in addition to protective effects on the skin against ultraviolet‐induced damage (Svobodová, Rambousková, Walterová, & Vostálová, 2008; Vostálová et al., 2013). However, it appears that more detailed studies are needed on the hepatoprotective effects of BH.

In the present study, we aimed to screen the efficacy of three different types of BH extracts in a mouse model of CCl_4_‐induced acute hepatic damage for their potential as potent hepatoprotective medicinal foods. The effects of the BH extracts were compared with those of silymarin (mixed flavonoids purified from milk thistle, *Silybum marianum*) (Jain, Lodhi, Jain, Nahata, & Singhai, 2011; Wang, Feng, Zhu, Zhao, & Suo, 2016).

## 2 MATERIALS AND METHODS

### 2.1 Preparation of BH extracts

Three different types of BH extract, BHw, BHj, and BHe, were prepared. BHw and BHj were prepared and supplied by Bioport Korea Inc. (Busan, Korea). BHw is the freeze‐dried powder of the hot water extract of BH; briefly, water and dried BH were mixed in a 10:1 (w/w) ratio and then boiled at 90–95°C for 3 h under reflux. The supernatant was condensed (55–65°C) and freeze dried. BHj is the freeze‐dried power of BH squeezed juice and BHe is the freeze‐dried powder of BH solution obtained after enzyme treatment. BH solution was purchased from H&K Bioscience Co. Ltd (Seoul, Korea) and freeze dried by Aribio Co. Ltd (Sungnam, Korea). Briefly, frozen BH fruits were treated by the following: heating (45–55°C for 3 min), pulverization, enzyme treatment (pectinase: Natuzyme DP ultra 0.05% (w/w), Natuzyme olimax 0.05% (w/w), 2–2.5 h, 50 rpm), centrifugation (6,400 *g*/min), heating (80°C, 15–30 s), addition of chitosan (0.005%, w/w) and guar gum (0.005% w/w), filtration (disc separation, diatomite filtration, filter press), condensation (63 brix, 50°C, 1 min, 0.092 MPa), sterilization (90–95°C, 15–30 s), and then freeze dried; from this process, BHe was obtained at a yield of 10.83%.

In addition, silymarin was purchased in the form of a reddish‐yellow powder from Sigma‐Aldrich Co. LLC. (St. Louis, MO, USA) and used as a reference drug (Jain et al., 2011; Wang et al., 2016).

All three different types of BH extracts were dissolved in distilled water to 20 mg/ml, and orally administered once per day for 7 days, consecutively. The administration volume was 10 ml/kg (equivalent to 200 mg/kg), which was applied through gastric gavage using a zonde attached to a 1 ml syringe. Silymarin was suspended into distilled water at 10 mg/ml, and orally administered in a volume of 10 ml/kg (equivalent 100 mg/kg) once per day for 7 days, consecutively, in accordance with the reference recommendation (Jain et al., 2011; Wang et al., 2016). In the intact vehicle and CCl_4_‐treated control mice, equal volumes of vehicle (distilled water) were orally administered instead of the test substances.

### 2.2 Animals and husbandry

A total of sixty, healthy male ICR mice (6 weeks old upon receipt from OrientBio, Seungnam, Korea) were used after acclimatization period of 7 days. Four or five animals were allocated to a polycarbonate cage in a temperature‐ (20–25°C) and humidity‐ (30–35%) controlled room. The light‐dark cycle was 12 h/12 h and the rats were given ad libitum access to feed (Cat. No. 38057; Purinafeed, Seungnam, Korea) and water were accessed.

**Figure 1:**
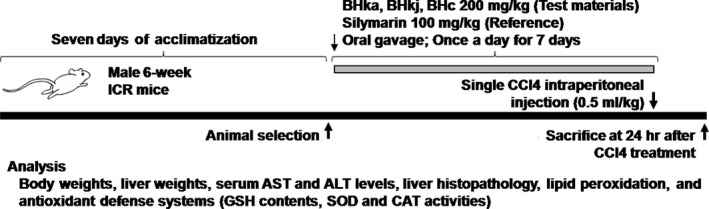
Experimental designs used in this study

The animals were divided into six groups based on their body weight prior to test substances administration: Intact control: Distilled water (DW) orally administered and olive oil intraperitoneally (IP) treated mice; CCl_4_ control: DW orally administered and CCl_4_ 0.5 mg/kg IP treated mice; Silymarin: Silymarin 100 mg/kg orally administered and CCl_4_ 0.5 ml/kg IP treated mice; BHw: BHw 200 mg/kg orally administered and CCl_4_ 0.5 ml/kg IP treated mice; BHj: BHj 200 mg/kg orally administered and CCl_4_ 0.5 ml/kg IP treated mice; and BHe: BHe 200 mg/kg orally administered and CCl_4_ 0.5 ml/kg IP treated mice (Figure 1).

### 2.3 Induction of acute liver damage

Acute liver damage was induced by single IP injection of CCl_4_ (Sigma‐Aldrich, St Louis, MO, USA), dissolved in olive oil (Sigma‐Aldrich, St Louis, MO, USA) 1:19 (v/v) (5%) in a volume of 10 ml/kg (equivalent to 0.5 ml/kg of CCl_4_), at 1 h after the seventh administration of the test material in accordance with previously established methods (Al‐Sayed, Abdel‐Daim, Kilany, Karonen, & Sinkkonen, 2015; Al‐Sayed et al., 2014; Fahmy, Al‐Sayed, Abdel‐Daim, Karonen, & Singab, 2016; Ferreira et al., 2010; Wang et al., 2013). Instead of CCl_4_, equal volumes of olive oils were administered to the intact control mice; further, distilled water was orally administered 1 h after the seventh administration.

### 2.4 Changes in body weights

Changes in body weight were measured each day, from 1 day before the administration of the initial test, throughout all experimental periods, by using an automatic electronic balance (Precisa Instrument, Zürich, Switzerland). To reduce the individual differences, the body weight gains from the day of initial test substance administration to 24 h after CCl_4_ injection were calculated as follows: Body weight gains (g) during 7 days of the whole experimental period = Body weight at 24 h after CCl_4_ treatment – Body weight on the day of initial test substance administration.

### 2.5 Measurements of liver weights

All animals were sacrificed 24 h after the CCl_4_ injection, gross inspection was conducted under anesthesia induced with 2–3% isoflurane (Hana Pharm. Co. Ltd, Hwasung, Korea) in a mixture of 70% N_2_O and 28.5% O_2_ using by using a rodent inhalation anesthesia apparatus (Surgivet, Waukesha, WI, USA) and rodent ventilator (Model 687, Harvard Apparatus, Cambridge, UK) and the weight of liver was measured (absolute wet‐weights). To reduce the differences from individual body weights, the relative liver weights (as a percentage of body weights) were also calculated from the following formula: relative liver weights (% of body weight) = (absolute liver wet‐weights/body weight at sacrifice) × 100.

### 2.6 Measurement of serum AST and ALT levels

At sacrifice, approximately 1 ml of venous blood was collected from the *vena cava*. All collected blood samples were centrifuged at 12,600 *g* for 10 min under cool temperatures (4°C) by using clotting activated serum tubes for serum separations and stored in an ultradeep freezer (Model MDF‐1156, Sanyo, Tokyo, Japan) below −150°C until analysis. Serum AST and ALT levels were detected by using an automated blood analyzer (Model Dri‐Chem NX500i, Fuji Medical System Co., Ltd, Tokyo, Japan).

### 2.7 Measurement of liver lipid peroxidation

The separated hepatic tissues were weighed and homogenized in ice‐cold 0.01 M Tris‐HCl buffer (pH 7.4) and centrifuged at 12,000 *g* for 15 min, as described by Kavutcu et al. (1996). Tissue homogenates were stored in an ultradeep freezer below −150°C until analysis. The concentration of liver lipid peroxidation was determined through the estimation of MDA by using the thiobarbituric acid test and a UV/Vis spectrophotometer (Model OPTIZEN POP, Mecasys, Daejeon, Korea) to measure the absorbance of the solution at 525 nm, and determined as nM of MDA/mg protein (Jamall & Smith, 1985). The total protein content was measured by a previously described method (Lowry, Rosenbrough, Farr, & Randall, 1951) using bovine serum albumin (Invitrogen, Carlsbad, CA, USA) as internal standard.

### 2.8 Measurement of hepatic antioxidant defense systems

The prepared hepatic homogenates were mixed with 0.1 ml 25% trichloroacetic acid (Merck, West Point, CA, USA) and centrifuged (1,627 *g*, 40 min, 4°C). The GSH content was spectrophotometrically determined through the measurement of absorbance at 412 nm by using 2‐nitrobenzoic acid (Sigma‐Aldrich, St. Louis, MO, USA) (Sedlak & Lindsay, 1968). The decomposition of H_2_O_2_ in the presence of CAT was followed at 240 nm by using a spectrophotometer (Aebi, 1974). CAT activity was defined as the amount of enzyme required to decompose 1 nM of H_2_O_2_ per minute at 25°C and pH 7.8. The results were expressed as U/mg protein. The measurement of SOD activity was performed in accordance with the method of Sun, Larry, and Ying (1988). SOD estimation was based on the generation of superoxide radicals produced by xanthine and xanthine oxidase, which react with nitrotetrazolium blue to produce formazan dye. SOD activity, which was related to the degree of inhibition of this reaction, was then spectrophotometrically measured at 560 nm and expressed as U/mg protein. One unit of SOD enzymatic activity is equal to the amount of enzyme that diminishes the initial absorbance of nitroblue tetrazolium by 50% over 1 min.

### 2.9 Histopathology

The left lateral lobes of the liver were separated and fixed in 10% neutral buffered formalin (NBF), embedded in paraffin, sectioned (3–4 μm), and stained with hematoxylin and eosin (H&E) for general histopathological analysis (Ki et al., 2013; Lee et al., 2014). The histopathological profiles of each sample were generated by observation under a light microscope (Eclipse 80*i*, Nikon, Tokyo, Japan). To observe more detailed changes, hepatic damage was evaluated by the modified HAI (histological activity index) grading scores based on a previous well‐established semiquantitative histopathological scoring system (Ishak et al., 1995), which includes assessment of confluent necrosis, focal lytic necrosis, apoptosis, and focal and portal inflammation (Table 1). In addition, the percentage of degenerative regions (%/mm^2^) in the liver that exhibited centrolobular necrosis, congestion, and inflammatory cell infiltrations on hepatic lobules was computed by using a software‐based automated image analyzer (*i*Solution FL ver 9.1, IMT *i*‐solution Inc., Vancouver, Quebec, Canada). The numbers of hepatocytes that showed degenerative changes, including necrosis, acute cellular swelling (ballooning), and severe fatty acid changes, and inflammatory cells infiltrated were also calculated by using an automated image analyzer and expressed as cells/1000 hepatocytes and cells/mm^2^ of hepatic parenchyma in accordance with previously described methods (Ki et al., 2013; Lee et al., 2014). The histopathologist was blinded to the group distribution when the analysis was performed.

**Table 1:** Modified HAI grading: inflammatory scores used in the present study

A. Confluent necrosis		
Absent	0	
Focal confluent necrosis		1
Zone 3 necrosis in some areas	2	
Zone 3 necrosis in most areas	3	
Zone 3 necrosis + occasional portal‐central bridging	4	
Zone 3 necrosis + multiple portal‐central bridging	5	
Panacinar or multiacinar necrosis		6
B. Focal (spotty) lytic necrosis, apoptosis and focal inflammation		
Absent	0	
One focus or less per 10× objective		1
Two to four foci per 10× objective		2
Five to ten foci per 10× objective		3
More than ten foci per 10× objective		4

HAI: Histological Activity Index; HAI grading scores = A + B; Possible maximum total scores = 10. Modified from the method described by Ishak et al. (1995).

### 2.10 Immunohistochemistry

The changes in apoptotic markers (cleaved caspase‐3 and PARP) (Jiang et al., 2012; Talwar et al., 2013; Yu et al., 2014), a marker of lipid peroxidation (4‐HNE) (Lee et al., 2014; Smathers, Galligan, Stewart, & Petersen, 2011), and an NO related oxidative stress marker (NT) (Lee et al., 2014; Pacher, Beckman, & Liaudet, 2007) were observed by the use of immunohistochemical staining methods by using purified primary antibodies (Table 2) with an ABC and peroxidase substrate kit (Vector Labs, Burlingame, CA, USA). Briefly, endogenous peroxidase activity was blocked by incubation in methanol and 0.3% H_2_O_2_ for 30 min and non‐specific binding of immunoglobulin was blocked through incubation with normal horse serum blocking solution for 1 h in a humidity chamber after heating (95–100°C); epitope retrievals were conducted in 10 mM citrate buffers (pH 6.0) (Ki et al., 2013; Lee et al., 2014; Yu et al., 2014). The primary antisera were treated overnight at 4°C in a humidity chamber and incubated with biotinylated universal secondary antibody and ABC reagents for 1 h at room temperature in humidity chamber. Finally, the sections were reacted with a peroxidase substrate kit for 3 min at room temperature. All sections were rinsed three times in 0.01 M PBS between each step. Cells that contained over 20% of immunoreactive staining by density, of cleaved caspase‐3, cleaved PARP, NT, and 4‐HNE, were regarded as positive in this study, and the numbers of cleaved caspase‐3, cleaved PARP, NT and 4‐HNE‐immunolabeled cells located within a restricted view field of hepatic parenchyma around centrolobular regions, around central veins as cells/1000 hepatocytes were measured by using a computer‐based automated image analyzer as previously described (Ki et al., 2013; Lee et al., 2014; Yu et al., 2014), respectively. The histopathologist was blinded to the group distribution when this analysis was performed.

**Table 2:** Primary antisera and detection kits for immunohistochemistry

Antisera or detection kits	Code	Source	Dilution
Primary antisera			
Anti‐cleaved caspase‐3 (Asp175) antibody	9661	Cell Signaling Technology Inc, Beverly, MA, USA	1:400
Anti‐cleaved PARP (h215) antibody	sc‐23461	Santa Cruz Biotechnology Inc, Santa Cruz, CA, USA	1:100
Anti‐Nitrotyrosine polyclonal antibody	06‐284	Millipore Corporation, Temecula, CA, USA	1:200
Anti‐4‐Hydroxynonenal polyclonal antibody	Ab46545	Abcam, Cambridge, UK	1:100
Detection kits			
Vectastain Elite ABC kit	PK‐6200	Vector Lab., Burlingame, CA, USA	1:50
Peroxidae substrate kit	SK‐4100	Vector Lab., Burlingame, CA, USA	1:50

All antiserum were diluted using 0.01 M phosphate buffered saline (pH 7.2). PARP: Poly(ADP‐ribose) polymerase.

### 2.11 Statistical analyses

All numerical data were expressed as the mean ± *SD* of 10 mice. Multiple comparison tests for different dose groups were conducted. Variance homogeneity was examined by using the Levene test (Levene, 1981). If the Levene test indicated no significant deviations from variance homogeneity, the obtain data were analyzed by one‐way ANOVA followed by a least‐significant differences (LSD) multi‐comparison test to determine which pairs of groups were significantly different. In the case that significant deviations from variance homogeneity were observed in the Levene test, a non‐parametric comparison test, the Kruskal‐Wallis *H* test, was conducted. When a significant difference was observed in the Kruskal‐Wallis *H* test, the Mann‐Whitney *U* (MW) test was conducted to determine the specific pairs of groups that are significantly different (Ludbrook, 1997). Differences were considered significant at *p *<* *0.05. Statistical analyses were computed by using SPSS for Windows (Release 14.0K, SPSS Inc., Chicago, IL, USA).

In addition, the percentage change between intact control mice and CCl_4_ control mice was calculated to evaluate the severity of hepatic damages, including the induction of centrolobular necrosis, and the percentage changes compared with the CCl_4_ control and BH‐ or silymarin‐treated mice were also calculated to help elucidate the efficacy of the test substances. These calculations were performed by Equations (1) and (2), respectively, in accordance with our previously established method (Kang et al., 2014).

(1) $$\begin{array}{cc} & \text{Percentage changes compared with intact control}\;\left(\%\right)=[((\text{Data of}\;{\text{CCl}}_{4}\;\text{control}\\ & -\text{Data of intact control mice})/\text{Data of intact control mice})\times 100] \end{array}$$

(2) $$\begin{array}{cc} & \text{Percentage changes compared with}\;{\text{CCl}}_{4}\;\text{control}\left(\%\right)=[((\text{Data of test substance treated mice}\\ & -\text{Data of}\;{\text{CCl}}_{4}\;\text{control mice})/\text{Data of}\;{\text{CCl}}_{4}\;\text{control mice})\times 100] \end{array}$$

## 3 RESULTS

### 3.1 Changes in body weight

**Figure 2:**
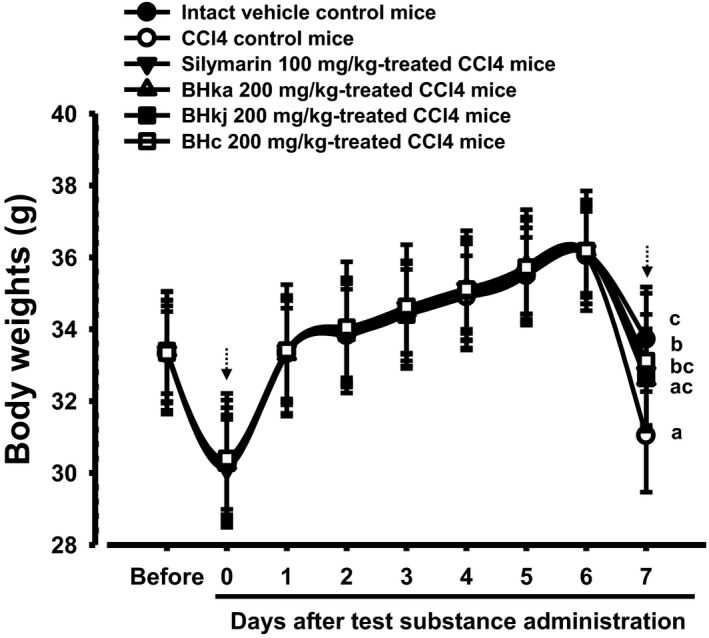
Body weights changes in intact or CCl_4_‐treated mice. Values are expressed mean ± *SD* of 10 mice. BH: Blue honeysuckle (Berries *of Lonicera caerulea* var. *edulis* L., Caprifoliaceae); BHw: lyophilized powder of BH aqueous extract; BHj: lyophilized powder of BH squeezed juice; BHe: lyophilized powder of BH enzyme extract. D‐1 means 1 day before first test substance administration. Day 7 means the day of sacrifice, 24 h after CCl_4_ treatment. All animals were overnight fasted before initial test substance administration and sacrifice (dot arrows). ^a^ *p* < 0.01 as compared with intact control by LSD test. ^b^ *p* < 0.01 and ^c^ *p* < 0.05 as compared with CCl_4_ control by LSD test

A significant (*p* < 0.01) decrease in body weight was detected on the day of sacrifice in the treated mice compared with the intact control; the body weight gain over the 7 day experimental period was also significantly (*p* < 0.01) decreased in the CCl_4_ control mice compared with that of the intact control mice. Significant (*p* < 0.01 or *p* < 0.05) increases in body weight at sacrifice and body weight gain during the 7 day experimental period were demonstrated in all mice administered each test substance in comparison with those administered the CCl_4_‐treated control. The order of change was as follows (largest to smallest): BHe, silymarin, BHj, and BHw (Table 3, Figure 2).

**Table 3:** Body weight gains in CCl_4_‐treated mice

Periods Groups	Body weights at				Body weight gains [B‐A]
	One day before test substance administration	First test substance administration [A]*	Last 7th test substance administration	24 h after CCl_4_ treatment [B]*	
Controls					
Intact	33.35 ± 1.14	30.24 ± 1.25	36.14 ± 1.14	33.72 ± 1.46	3.48 ± 0.73
CCl_4_	33.32 ± 1.33	30.33 ± 1.49	36.05 ± 1.34	31.04 ± 1.57^a^	0.71 ± 1.14^a^
Silymarin	33.37 ± 1.43	30.13 ± 1.48	36.18 ± 1.27	32.79 ± 1.62^d^	2.66 ± 0.91^c^
BH extracts					
BHw	33.40 ± 1.66	30.26 ± 1.77	36.19 ± 1.30	32.60 ± 1.28^d^	2.34 ± 0.95^ac^
BHj	33.40 ± 1.42	30.28 ± 1.53	36.15 ± 1.45	32.64 ± 1.37^d^	2.36 ± 1.27^bc^
BHe	33.34 ± 1.70	30.40 ± 1.81	36.18 ± 1.67	33.12 ± 1.88^c^	2.72 ± 0.41^c^

Values are expressed mean ± *SD* of 10 mice, g. BH: Blue honeysuckle (Berries *of Lonicera caerulea* var. *edulis* L., Caprifoliaceae); BHw: lyophilized powder of BH aqueous extract; BHj: lyophilized powder of BH squeezed juice; BHe: lyophilized powder of BH enzyme extract.

*All animals were overnight fasted (about 18 h; water was not restrict). ^a^ *p* < 0.01 and ^b^ *p* < 0.05 as compared with intact control by LSD test. ^c^ *p* < 0.01 and ^d^ *p* < 0.05 as compared with CCl_4_ control by LSD test.

The body weight gains during the 7 day whole experimental period in the CCl_4_ control were found to be −79.60% compared with intact control, but the values in silymarin 100 mg/kg and BHw, BHj, and BHe 200 mg/kg treated mice were 274.65%, 229.58%, 232.39%, and 283.10%, respectively, compared with the CCl_4_ control.

### 3.2 Changes on gross appearance and liver weights

**Figure 3:**
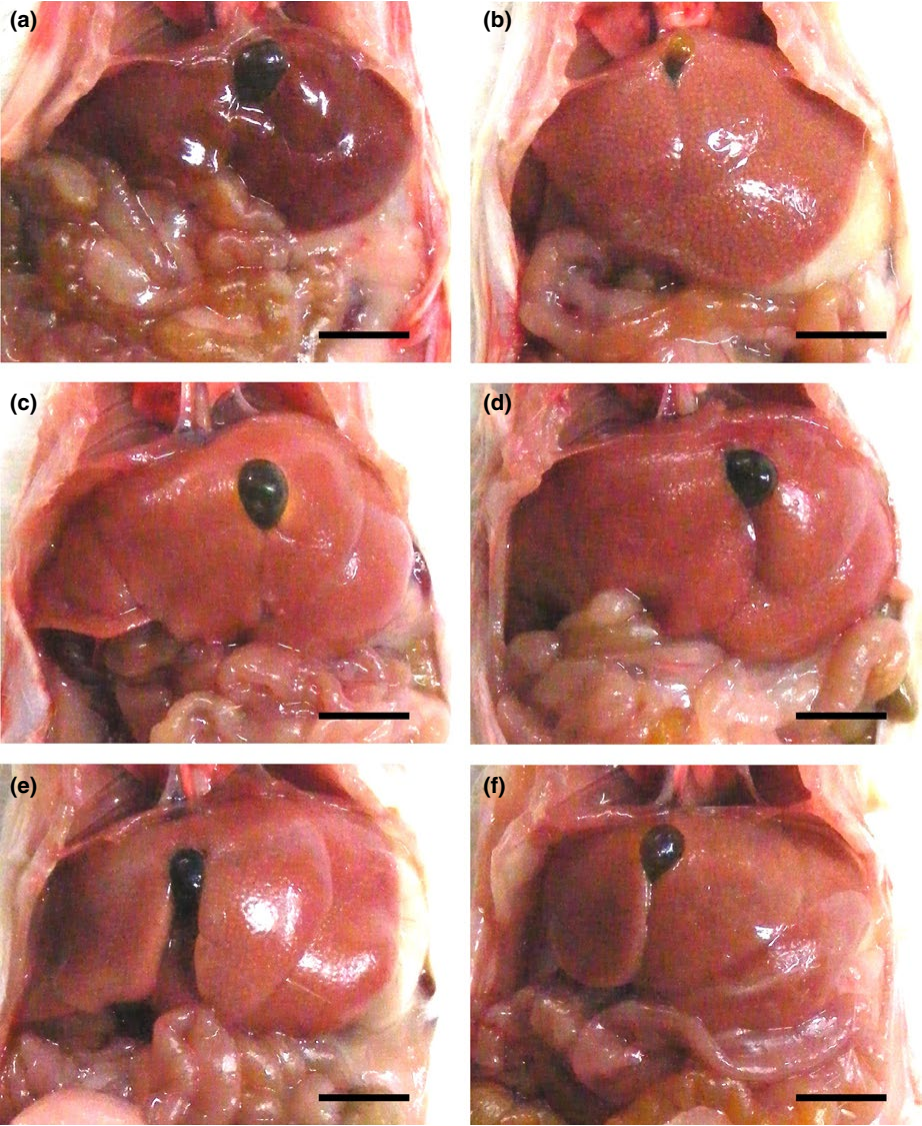
Representative gross liver images, taken from intact or CCl_4_‐treated mice. (a) Intact control (Distilled water and olive oil treated mice). (b) CCl_4_ control (Distilled water and CCl_4_ 0.5 ml/kg treated mice). (c) Silymarin control (Silymarin 100 mg/kg and CCl_4_ 0.5 ml/kg treated mice). (d) BHw (BHw 200 mg/kg and CCl_4_ 0.5 ml/kg treated mice). (e) BHj (BHj 200 mg/kg and CCl_4_ 0.5 ml/kg treated mice). (f) BHe (BHe 200 mg/kg and CCl_4_ 0.5 ml/kg treated mice). BH: Blue honeysuckle (Berries *of Lonicera caerulea* var. *edulis* L., Caprifoliaceae); BHw: lyophilized powder of BH aqueous extract, BHj: lyophilized powder of BH squeezed juice; BHe: lyophilized powder of BH enzyme extract; CCl_4_: Carbone tetrachloride. Scale bars: 6.5 mm

**Figure 4:**
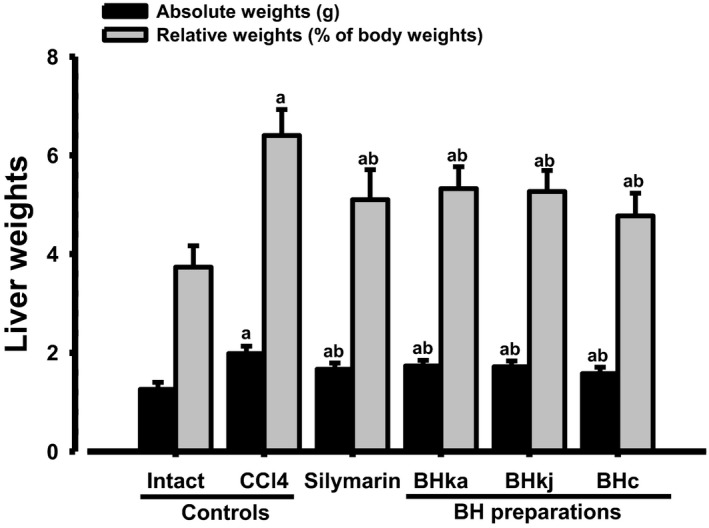
Liver weights in the CCl_4_‐treated mice. Values are expressed mean ± SD of 10 mice. BH: Blue honeysuckle (Berries *of Lonicera caerulea* var. *edulis* L., Caprifoliaceae); BHw: lyophilized powder of BH aqueous extract; BHj: lyophilized powder of BH squeezed juice; BHe: lyophilized powder of BH enzyme extract. ^a^ *p* < 0.01 as compared with intact control by LSD test. ^b^ *p* < 0.01 as compared with CCl_4_ control by LSD test

Marked small nodule formation and the enlargement of the liver were demonstrated in the CCl_4_ control compared with the intact control upon gross inspection, with related significant (*p* < 0.01) increases in the absolute and relative weights of the liver. However, noticeable decreases in small nodule formations and hepatic enlargements, and related significant (*p* < 0.01) decreases of liver weights were observed in all mice administered the test substance compared with the CCl_4_ control. The induced changes occurred in the following order (largest to smallest): BHe, silymarin, BHj, and BHw (Figures 3 and 4).

The absolute liver weight in the CCl_4_ control was changed by 57.75% compared with the intact control, but this was ameliorated by the administration of silymarin 100 mg/kg, BHw, BHj, and BHe 200 mg/kg to −16.09%, −12.67%, −13.49%, and −20.56%, respectively, compared with the CCl_4_ control. The relative liver weights in the CCl_4_ control were 71.50% of the intact control, but were altered by the treatment of silymarin 100 mg/kg, BHw, BHj, and BHe 200 mg/kg to −20.31%, −16.84%, −17.74%, and −25.48%, respectively, compared with the CCl_4_ control.

### 3.3 Changes in the serum AST and ALT levels

**Figure 5:**
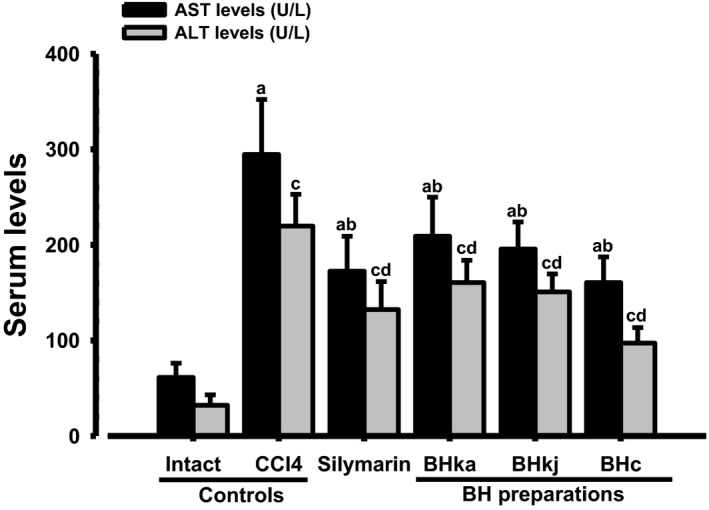
Serum AST and ALT levels in the CCl_4_‐induced liver damaged mice. Values are expressed mean ± SD of 10 mice. BH: Blue honeysuckle (Berries *of Lonicera caerulea* var. *edulis* L., Caprifoliaceae); BHw: lyophilized powder of BH aqueous extract; BHj: lyophilized powder of BH squeezed juice; BHe: lyophilized powder of BH enzyme extract; AST: Aspartate aminotransferase; ALT: Alanine aminotransferase. ^a^ *p* < 0.01 as compared with intact control by LSD test. ^b^ *p* < 0.01 as compared with CCl_4_ control by LSD test. ^c^ *p* < 0.01 as compared with intact control by MW test. ^d^ *p* < 0.01 as compared with CCl_4_ control by MW test

Significant (*p* < 0.01) elevations of serum AST and ALT levels (intracellular enzymes indicative of hepatic damages) were observed in the CCl_4_ control compared with the intact control, but significant (*p* < 0.01) decreases were induced by the treatment of all BH extracts at 200 mg/kg, and by silymarin 100 mg/kg, compared with the CCl_4_ control. The changes were largest in BHe, followed by silymarin, BHj, and BHw (Figure 5).

The serum AST levels in the CCl_4_ control were increased to 380.59% compared with the intact control, but were found to be decreased by silymarin 100 mg/kg, BHw, BHj, and BHe 200 mg/kg to −41.48%, −29.06%, −33.60%, and −45.52%, respectively, compared with the CCl_4_ control. The serum ALT levels in the CCl_4_ control were increased to 584.42% compared with the intact control, but they were decreased by silymarin 100 mg/kg, BHw, BHj, and BHe 200 mg/kg to −39.78%, −26.95%, −31.36%, and −55.76%, respectively, compared with the CCl_4_ control.

### 3.4 Effects on the hepatic lipid peroxidation

Significant (*p* < 0.01) increases in hepatic lipid peroxidation and MDA content were found in the CCl_4_ control compared with the intact control. However, significant (*p* < 0.01) decreases in MDA content were induced by all BH extracts at 200 mg/kg and silymarin 100 mg/kg compared with the CCl_4_ control; the largest changes were induced by BHe, followed by silymarin, BHj, and BHw (Table 4).

**Table 4:** Hepatic lipid peroxidation, GSH contents, and CAT and SOD activities in CCl_4_‐treated mice

Items (Unit) Groups	Lipid Peroxidation (nM of MDA/mg protein)	GSH Contents (nM/mg protein)	Enzyme activity	
			SOD (U/mg protein)	CAT (U/mg protein)
Controls				
Intact	1.49 ± 0.86	38.48 ± 10.33	418.83 ± 135.08	242.68 ± 102.48
CCl_4_	8.31 ± 1.44^a^	3.78 ± 1.21^c^	58.76 ± 27.74^c^	45.60 ± 18.65^c^
Silymarin	5.00 ± 1.13^ab^	17.59 ± 3.71^cd^	184.74 ± 72.99^cd^	121.85 ± 22.91^cd^
BH extracts				
BHw	6.05 ± 1.10^ab^	10.74 ± 2.89^cd^	126.68 ± 35.63^cd^	88.15 ± 21.04^cd^
BHj	5.54 ± 1.12^ab^	12.51 ± 2.50^cd^	145.29 ± 39.18^cd^	104.39 ± 20.66^cd^
BHe	4.45 ± 1.45^ab^	21.41 ± 5.08^cd^	223.66 ± 55.75^cd^	133.21 ± 20.74^cd^

Values are expressed mean ± *SD* of 10 mice. BH: Blue honeysuckle (Berries *of Lonicera caerulea* var. *edulis* L., Caprifoliaceae); BHw: lyophilized powder of BH aqueous extract, BHj: lyophilized powder of BH squeezed juice, BHe: lyophilized powder of BH enzyme extract; MDA: Malondialdehyde; CAT: Catalase; SOD: Superoxide dismutase.

^a^ *p* < 0.01 as compared with intact control by LSD test. ^b^ *p* < 0.01 as compared with CCl_4_ control by LSD test. ^c^ *p* < 0.01 as compared with intact control by MW test. ^d^ *p* < 0.01 as compared with CCl_4_ control by MW test.

The hepatic MDA contents in the CCl_4_ control were increased to 457.65% compared with the intact control, but were decreased by silymarin 100 mg/kg, BHw, BHj, and BHe 200 mg/kg to −39.78%, −27.19%, −33.34%, and −46.48%, respectively, compared with the CCl_4_ control.

### 3.5 Effects on hepatic GSH, an endogenous antioxidant

A significant (*p* < 0.01) decrease of hepatic GSH was observed in the CCl_4_ control mice compared with the intact control, but these changes were significantly (*p* < 0.01) ameliorated by oral pre‐administration for 7 days for all BH extracts at 200 mg/kg and by silymarin 100 mg/kg compared with the CCl_4_ control. BHe exerted the strongest effect, followed by silymarin, BHj, and BHw (Table 4).

The hepatic GSH contents in the CCl_4_ control were changed to −90.17% compared with the intact control, but they were increased by silymarin 100 mg/kg, BHw, BHj, and BHe 200 mg/kg to 364.72%, 183.85%, 230.58%, and 465.70%, respectively, compared with the CCl_4_ control.

### 3.6 Changes in activity of CAT, an endogenous antioxidative enzyme

A significant (*p* < 0.01) decrease in hepatic CAT activity was observed in the CCl_4_ control compared with the intact control, but the decrease induced by CCl_4_ was significantly (*p* < 0.01) ameliorated by the 7 day pre‐treatment of silymarin 100 mg/kg and all BH extracts at 200 mg/kg; BHe exerted the strongest effect, followed by silymarin, BHj, and BHw (Table 4).

The hepatic CAT activity in the CCl_4_ control was decreased to −81.21% compared with the intact control, but was increased by silymarin 100 mg/kg, BHw, BHj, and BHe 200 mg/kg to 167.20%, 93.31%, 128.92%, and 192.13% compared with the CCl_4_ control, respectively.

### 3.7 Effects on activity of SOD, another endogenous antioxidative enzyme

A significant (*p* < 0.01) decrease in hepatic SOD activity was detected in the CCl_4_ control compared with the intact control. However, significant (*p* < 0.01) increases in SOD activities occurred in mice that received pre‐treatment with all BH extracts at 200 mg/kg and silymarin 100 mg/kg compared with that of the CCl_4_ control. BHe exerted the strongest effect, followed by BHe, silymarin, BHj, and BHw (Table 4).

The hepatic SOD activity in the CCl_4_ control was decreased to −85.97% compared with the intact control, but was increased by silymarin 100 mg/kg, BHw, BHj, and BHe 200 mg/kg to 214.39%, 115.59%, 147.26%, and 280.62%, respectively, compared with the CCl_4_ control.

### 3.8 Histopathological inspection

**Figure 6:**
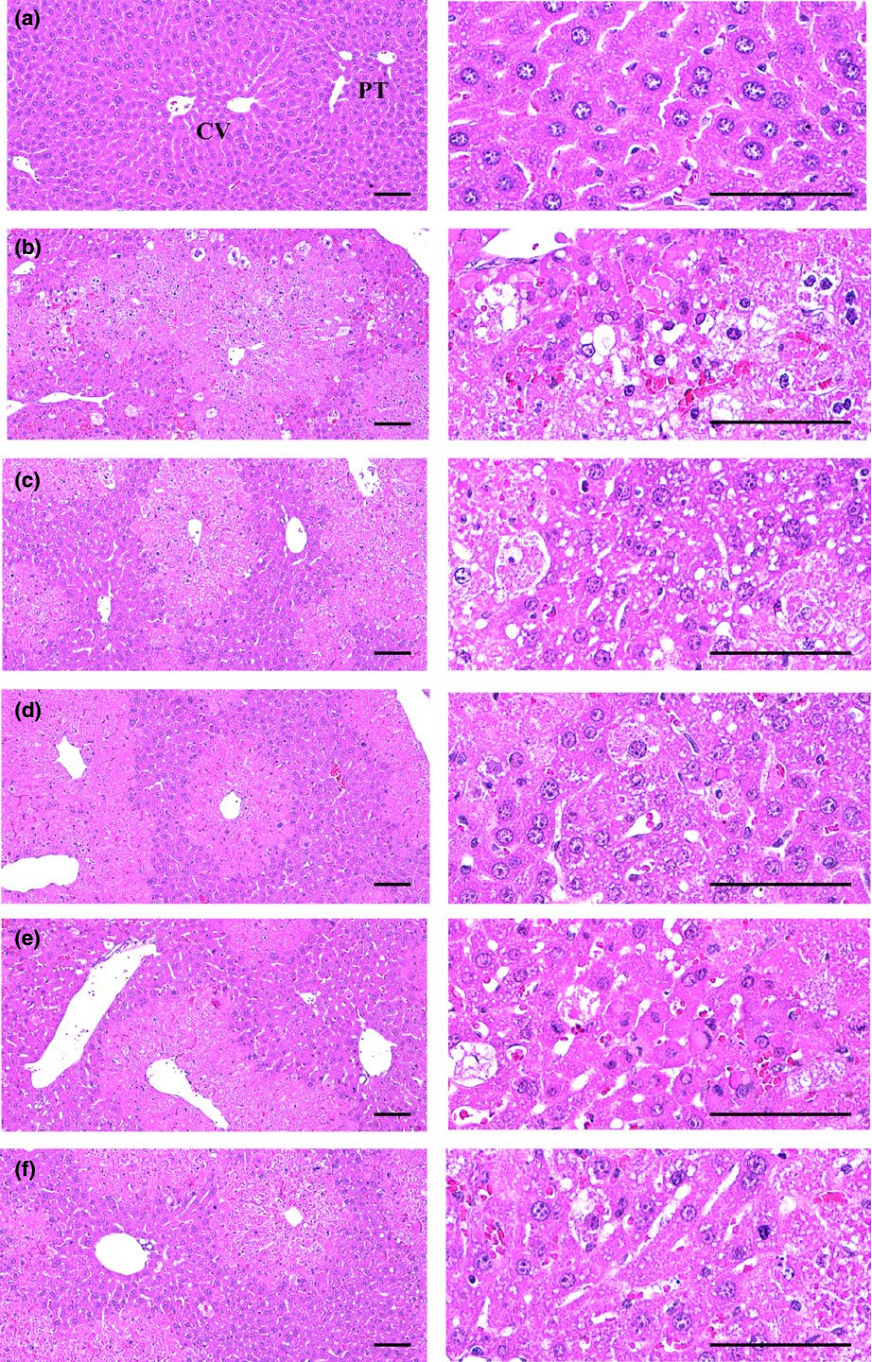
Histopathogical profiles of the CCl_4_ damaged liver. (a) Intact control (Distilled water and olive oil treated mice). (b) CCl_4_ control (Distilled water and CCl_4_ 0.5 ml/kg treated mice). (c) Silymarin control (Silymarin 100 mg/kg and CCl_4_ 0.5 ml/kg treated mice). (d) BHw (BHw 200 mg/kg and CCl_4_ 0.5 ml/kg treated mice). (e) BHj (BHj 200 mg/kg and CCl_4_ 0.5 ml/kg treated mice). (f) BHe (BHe 200 mg/kg and CCl_4_ 0.5 ml/kg treated mice). BH: Blue honeysuckle (Berries *of Lonicera caerulea* var. *edulis* L., Caprifoliaceae); BHw: lyophilized powder of BH aqueous extract; BHj: lyophilized powder of BH squeezed juice; BHe: lyophilized powder of BH enzyme extract; CV: Central vein; PT: Portal triad regions. All Hematoxylin‐eosin stain. Scale bars = 120 μm

Classic centrolobular necrosis (hepatocyte vacuolation and ballooning, deposition of lipid droplets in hepatocytes, and infiltration of inflammatory cells) was observed after a single IP treatment of CCl_4_ 0.5 ml/kg. However, this microscopic centrolobular necrosis was markedly inhibited by 7 days of continuous oral pre‐treatment of silymarin 100 mg/kg and by all BH extracts at 200 mg/kg compared with the CCl_4_ control; BHe was the most effective, followed by silymarin, BHj, and BHw. The histomorphometrical and semi‐quantitative analysis indicated significant (*p* < 0.01) increases in the percentage area of degenerative regions, the numbers of degenerative hepatocytes, and the numbers of inflammatory cells infiltrated in hepatic parenchyma; therefore, a related increase of modified HAI grading scores was observed in the CCl_4_ control compared with the intact control. These changes were significantly (*p* < 0.01) ameliorated by treatment with all BH extracts at 200 mg/kg and by silymarin 100 mg/kg compared with the CCl_4_ control; BHe exerted the strongest effect, followed by silymarin, BHj, and BHw (Table 5, Figure 6).

**Table 5:** General histomorphometrical analysis of hepatic tissues from CCl_4_‐treated mice

Items (Unit) Groups	General histomorphometry			
	Histological Activity Index (Scores; Max = 10)	Percentages of degenerative regions (%/mm^2^)	Numbers of degenerative hepatocytes (cells/1000 hepatocytes)	Numbers of inflammatory cells infiltrated (cells/mm^2^)
Controls				
Intact	0.40 ± 0.52	2.53 ± 1.95	29.60 ± 19.41	43.40 ± 16.60
CCl_4_	8.40 ± 1.07^a^	79.82 ± 10.06^c^	809.50 ± 100.54^c^	269.10 ± 74.04^c^
Silymarin	4.40 ± 0.84^ab^	44.22 ± 11.44^cd^	446.90 ± 108.91^cd^	73.50 ± 15.86^cd^
BH extracts				
BHw	5.00 ± 1.33^ab^	54.92 ± 12.47^cd^	563.30 ± 136.44^cd^	102.20 ± 25.42^cd^
BHj	4.80 ± 1.14^ab^	50.21 ± 11.52^cd^	496.10 ± 122.23^cd^	89.40 ± 21.54^cd^
BHe	3.50 ± 1.27^ab^	38.32 ± 11.02^cd^	407.40 ± 121.06^cd^	62.40 ± 23.80^d^

Values are expressed mean ± *SD* of 10 mice. BH: Blue honeysuckle (Berries *of Lonicera caerulea* var. *edulis* L., Caprifoliaceae); BHw: lyophilized powder of BH aqueous extract; BHj: lyophilized powder of BH squeezed juice; BHe: lyophilized powder of BH enzyme extract.

^a^ *p* < 0.01 as compared with intact vehicle control by LSD test. ^b^ *p* < 0.01 as compared with CCl_4_ control by LSD test. ^c^ *p* < 0.01 as compared with intact vehicle control by MW test. ^d^ *p* < 0.01 as compared with CCl_4_ control by MW test.

The percentage of degenerative regions in the CCl_4_ control was increased to 3053.58% compared with the intact control, but was decreased by silymarin 100 mg/kg, BHw, BHj, and BHe 200 mg/kg to −44.59%, −31.20%, −37.09%, and −51.99%, respectively, compared with the CCl_4_ control. The mean number of degenerated hepatocytes in the CCl_4_ control was increased to 2634.80% compared with the intact control, but decreased by silymarin 100 mg/kg, BHw, BHj, and BHe 200 mg/kg to −44.79%, −30.41%, −38.72%, and −49.67%, respectively, compared with the CCl_4_ control. The mean number of inflammatory cells infiltrated in the hepatic parenchyma of the CCl_4_ control was increased to 520.05% compared with the intact control, but was decreased by silymarin 100 mg/kg, BHw, BHj, and BHe 200 mg/kg to −72.69%, −62.39%, −66.78%, and −76.81%, respectively, compared with the CCl_4_ control. The mean modified HAI grading score in the CCl_4_ control was increased to 2000.00% compared with the intact control, but decreased by silymarin 100 mg/kg, BHw, BHj, and BHe 200 mg/kg to −47.62%, −40.48%, −42.86%, and −58.33%, respectively, compared with the CCl_4_ control.

### 3.9 Immunohistochemical analysis

**Figure 7:**
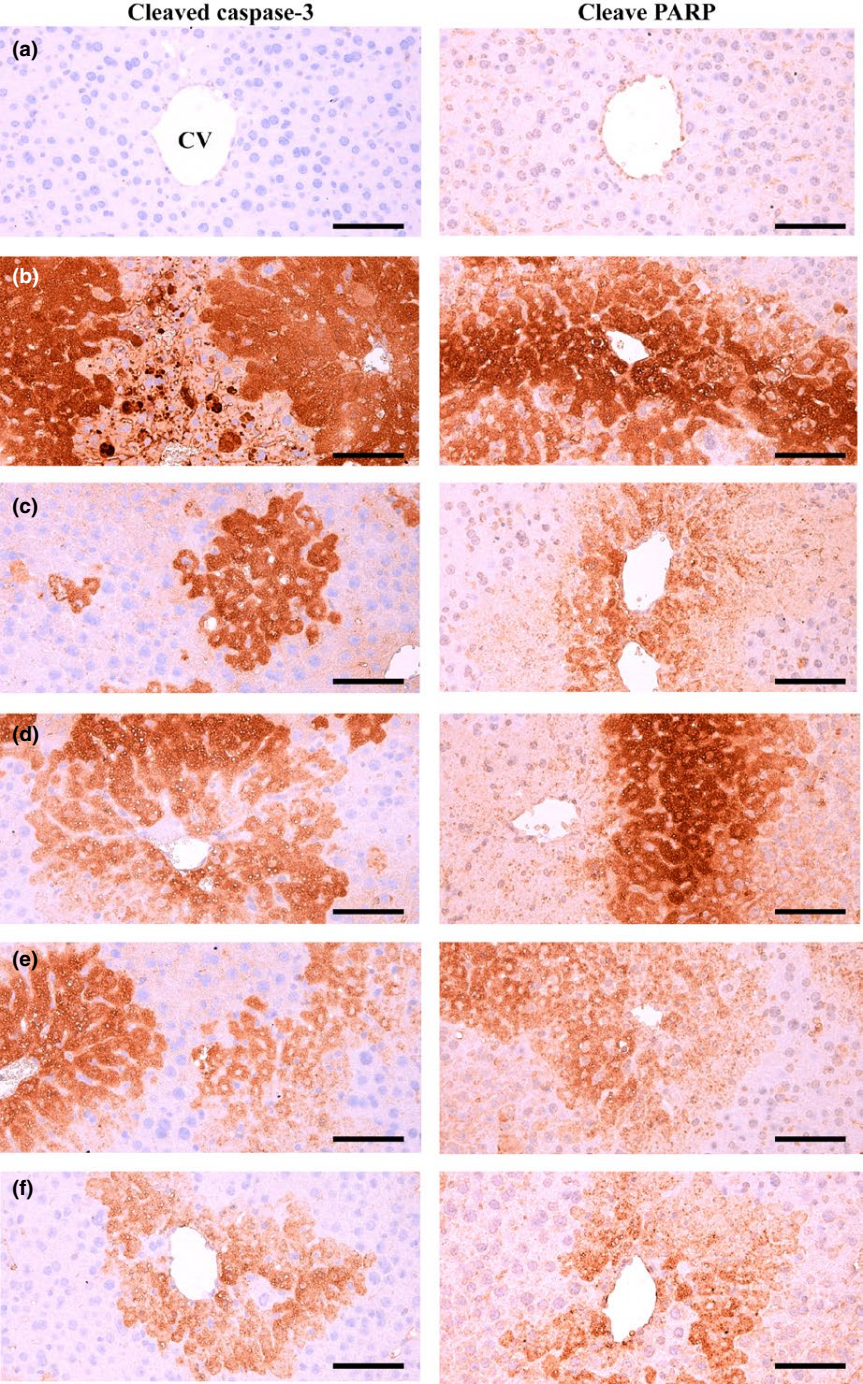
Cleaved caspase‐3 and PARP immunoreactivities in the CCl_4_ damaged liver. (a) Intact control (Distilled water and olive oil treated mice). (b) CCl_4_ control (Distilled water and CCl_4_ 0.5 ml/kg treated mice). (c) Silymarin control (Silymarin 100 mg/kg and CCl_4_ 0.5 ml/kg treated mice). (d) BHw (BHw 200 mg/kg and CCl_4_ 0.5 ml/kg treated mice). (e) BHj (BHj 200 mg/kg and CCl_4_ 0.5 ml/kg treated mice). (f) BHe (BHe 200 mg/kg and CCl_4_ 0.5 ml/kg treated mice). BH: Blue honeysuckle (Berries *of Lonicera caerulea* var. *edulis* L., Caprifoliaceae); BHw: lyophilized powder of BH aqueous extract; BHj: lyophilized powder of BH squeezed juice; BHe: lyophilized powder of BH enzyme extract; CV: Central vein; PARP: Poly(ADP‐ribose) polymerase. Immunoreactive cells were stained by avidin‐biotin‐peroxidase methods. Scale bars = 120 μm

**Figure 8:**
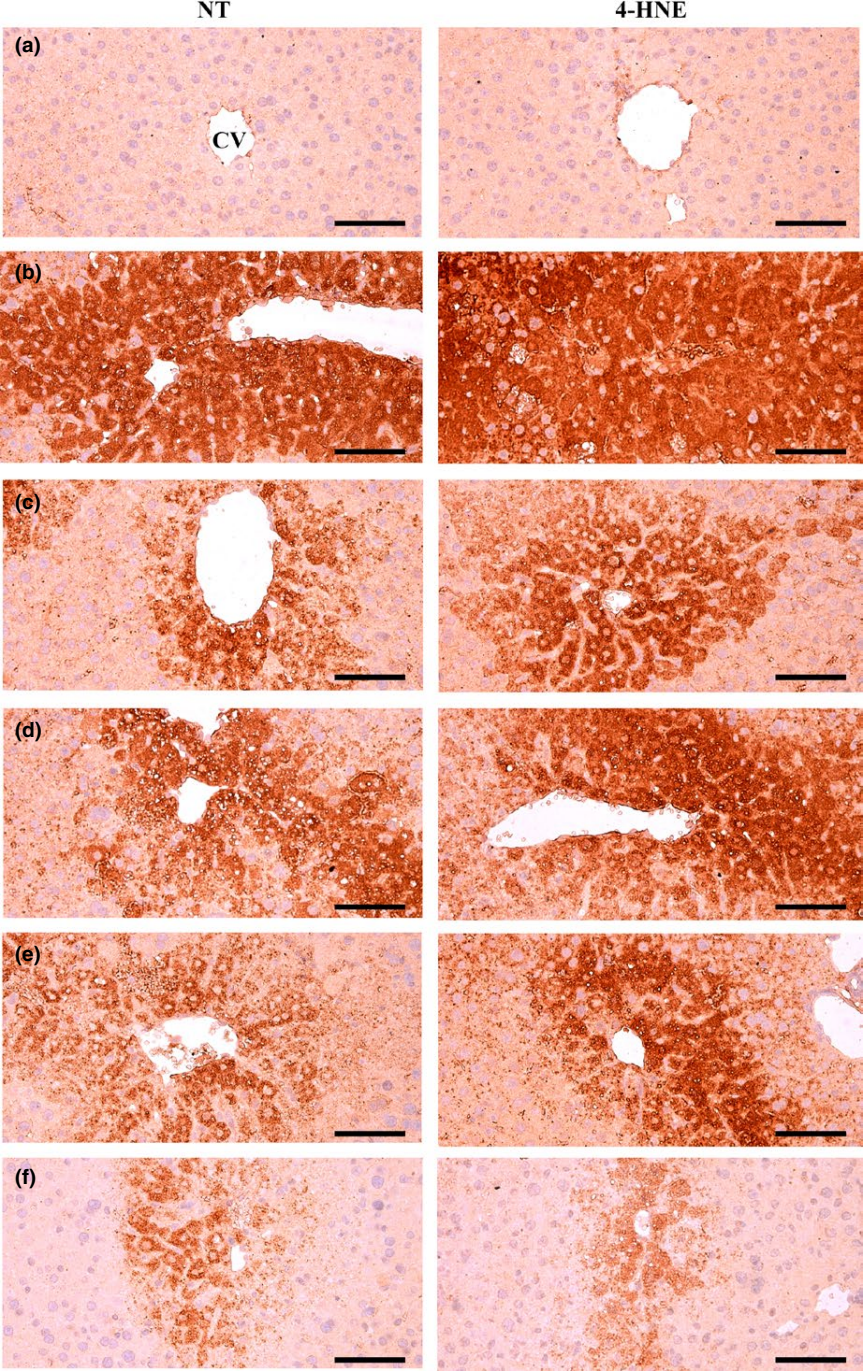
NT and 4‐HNE Immunoreactivities in the CCl_4_ induced damaged liver. (a) Intact control (Distilled water and olive oil treated mice). (b) CCl_4_ control (Distilled water and CCl_4_ 0.5 ml/kg treated mice). (c) Silymarin control (Silymarin 100 mg/kg and CCl_4_ 0.5 ml/kg treated mice). (d) BHw (BHw 200 mg/kg and CCl_4_ 0.5 ml/kg treated mice). (e) BHj (BHj 200 mg/kg and CCl_4_ 0.5 ml/kg treated mice). (f) BHe (BHe 200 mg/kg and CCl_4_ 0.5 ml/kg treated mice). BH: Blue honeysuckle (Berries *of Lonicera caerulea* var. *edulis* L., Caprifoliaceae); BHw: lyophilized powder of BH aqueous extract, BHj: lyophilized powder of BH squeezed juice, BHe: lyophilized powder of BH enzyme extract; CV: Central vein; NT: Nitrotyrosine; 4‐HNE: 4‐Hydroxynonenal. Immunoreactive cells were stained by avidin‐biotin‐peroxidase methods. Scale bars = 120 μm

Significant (*p* < 0.01) increases in the number immunoreactive hepatocytes to apoptotic markers (cleaved caspase‐3 and PARP), a marker of lipid peroxidation (4‐HNE) and an NO‐related oxidative stress marker (NT) were observed in the CCl_4_ control compared with the intact control by using ABC‐based immunohistochemistry. However, significant (*p* < 0.01) decreases of the mean numbers of cleaved caspase‐3, PARP, NT, and 4‐HNE immunoreactive hepatocytes were induced by all three types of BH extracts at 200 mg/kg and by silymarin 100 mg/kg compared with the CCl_4_ control; BHe exerted the strongest effect, followed by silymarin, BHj, and BHw (Table 6, Figures 7 and 8).

**Table 6:** Immunohistochemistrical‐histomorphometrical analysis of hepatic tissues from CCl_4_‐treated mice

Items (Unit) Groups	Positive cells by immunohistochemistry (cells/1000 hepatocytes)			
	Cleaved caspase‐3	Cleaved Poly(ADP‐ribose) polymerase	Nitrotyrosine	4‐Hydroxynonenal
Controls				
Intact	2.00 ± 1.41	4.10 ± 2.88	17.20 ± 10.12	15.80 ± 8.38
CCl_4_	718.20 ± 108.68^a^	714.40 ± 111.79^c^	707.10 ± 101.38^c^	757.10 ± 120.07^c^
Silymarin	383.00 ± 104.97^ab^	288.80 ± 64.00^cd^	331.50 ± 115.94^cd^	440.20 ± 135.73^cd^
BH extracts				
BHw	488.90 ± 119.27^ab^	470.40 ± 126.21^cd^	486.90 ± 108.44^cd^	535.70 ± 107.30^cd^
BHj	433.20 ± 107.51^ab^	454.80 ± 96.07^cd^	442.10 ± 66.79^cd^	480.90 ± 112.66^cd^
BHe	310.10 ± 111.16^ab^	196.90 ± 62.99^cd^	291.60 ± 69.14^cd^	264.10 ± 70.92^cd^

Values are expressed mean ± *SD* of 10 mice. BH: Blue honeysuckle (Berries *of Lonicera caerulea* var. *edulis* L., Caprifoliaceae); BHw: lyophilized powder of BH aqueous extract; BHj: lyophilized powder of BH squeezed juice; BHe: lyophilized powder of BH enzyme extract.

^a^ *p* < 0.01 as compared with intact vehicle control by LSD test. ^b^ *p* < 0.01 as compared with CCl_4_ control by LSD test. ^c^ *p* < 0.01 as compared with intact vehicle control by MW test. ^d^ *p* < 0.01 as compared with CCl_4_ control by MW test.

The mean number of cleaved caspase‐3 immunoreactive hepatocytes in the CCl_4_ control was increased to 35810.00% compared with the intact control, but was decreased by silymarin 100 mg/kg, BHw, BHj, and BHe 200 mg/kg to −46.67%, −31.93%, −39.68%, and −56.82%, respectively, compared with the CCl_4_ control. The mean number of PARP immunopositive hepatocytes in the CCl_4_ control was increased to 17324.39% compared with the intact control, but was decreased by silymarin 100 mg/kg, BHw, BHj, and BHe 200 mg/kg to −59.57%, −34.15%, −36.34%, and −72.44%, respectively, compared with the CCl_4_ control. The mean number of NT immunolabeled hepatocytes in the CCl_4_ control was increased to 4011.05% compared with the intact control, but was decreased by silymarin 100 mg/kg, BHw, BHj, and BHe 200 mg/kg to −53.12%, −31.14%, −37.48%, and −58.76% compared with the CCl_4_ control, respectively. The mean number of 4‐HNE immunostained hepatocytes in the CCl_4_ control was increased to 4691.77% compared with the intact control, but was decreased by silymarin 100 mg/kg, BHw, BHj, and BHe 200 mg/kg to −41.86%, −29.24%, −36.48%, and −65.12%, respectively, compared with the CCl_4_ control.

## 4 DISCUSSION

The common industrial solvent, CCl_4_, is one of the most potent inducers of acute liver injury; it is often used in animal studies to model human liver injury (Yang et al., 2015; Zou et al., 2016). As the liver is a vital organ with known importance in several physiological processes (Lu et al., 2016; Yang et al., 2015), the need for hepatoprotective medicines has gradually emerged. Modern medicine is still hampered by a lack of reliable hepatoprotective drugs; therefore, numerous traditional herbal medicines have been studied for their hepatoprotective efficiency (Lu et al., 2016). BH is a rich source of ascorbic acid and phenolic components, particularly anthocyanins, flavonoids, and low molecular weight phenolic acids, which exert multiple biological activities, including strong antioxidant activity (Chaovanalikit et al., 2004; Svarcova et al., 2007). BH extracts were shown to have the strongest antioxidant potential among 12 types of colored berries (Chen et al., 2014) and possess hepatoprotective effects (Palíková et al., 2009). However, as more detailed studies on these hepatoprotective effects are lacking, we aimed to evaluated the effect of BH extracts against CCl_4_‐induced acute hepatic damage in mice (Ferreira et al., 2010; Wang et al., 2013); we examined three different extract types, BHw, BHj, and BHe, for their suitability as potent hepatoprotective medicinal foods.

The decreased body weight after the administration of CCl_4_ is considered to result from the direct toxicity of CCl_4_ and/or indirect toxicity related to the liver damage; hence, the change in body weight after CCl_4_ treatment has been used as a valuable index in the efficacy test of CCl_4_‐related organ damage (Pradeep, Mohan, Anand, & Karthikeyan, 2005; Yang, Li, Wang, & Wu, 2010). All mice in the intact control group in this study experienced normal increases in body weight within the range of normal age‐matched mice of the same strain (Fox, Cohen, & Loew, 1984; Tajima, 1989). In the current study, a significant decrease in body weight was detected up to the day of sacrifice day (24 h after CCl_4_ treatment) compared with those of the intact control; accordingly, the body weight gains during the 7 day experimental period were also significantly decreased in the CCl_4_ control compared with those of the intact control. However, significant increases in the body weight at sacrifice and the body weight gain during the 7 day experimental period were demonstrated in all mice treated with the test substances compared with the CCl_4_ control. The strongest effects were exerted by BHe, followed by silymarin, BHj, and BHw. These findings were considered reliable evidence that all three BH extracts exerted favorable inhibitory effects on the CCl_4_‐induced body weight changes; the strongest effects were exerted by BHe, followed by BHj and BHw. In particular, BHe 200 mg/kg showed greater inhibition of the CCl_4_‐induced changes in body weights than that caused by silymarin 100 mg/kg.

It was observed that marked small nodulation and enlargement occurred in CCl_4_‐treated livers with related increases in liver weights (Han et al., 2016; Pinto, Duque, Rodríguez‐Galdón, Cestero, & Macías, 2012), but was also present in the CCl_4_ control. However, a noticeable decrease in small nodule formations and hepatic enlargements, with a related significant decrease in liver weights were observed in all mice administered test substances compared with those of the CCl_4_ control; BHe exerted the most favorable effect, followed by silymarin, BHj, and BHw. These findings were also considered to present clear evidence that all three BH extracts tested in this study induced favorable hepatoprotective effects on CCl_4_‐induced acute liver injury in mice. In particular, BHe 200 mg/kg showed more favorable inhibitory effects on the CCl_4_‐induced nodulation and enlargement of the liver with related increases of liver weights in mice compared with those of silymarin 100 mg/kg.

Generally, AST and ALT (formerly known as SGOT and SGPT) have been used as serum markers to represent liver damage (Sodikoff, 1995); these enzymes are markedly elevated in CCl_4_‐induced hepatic damage (Lu et al., 2016; Yang et al., 2015) and were elevated in the CCl_4_ control in the present study. Therefore, the results of our study provided further evidence that all three BH extracts tested in this study exerted favorable hepatoprotective effects against CCl_4_‐induced liver injuries; the strongest effect was induced by BHe, followed by BHj and BHw, as demonstrated by the marked and significant inhibitions on the CCl_4_‐induced serum AST and ALT elevations in these groups compared with the CCl_4_ control. Better inhibition of the CCl_4_‐induced elevation of serum AST and ALT were observed in mice treated with BHe 200 mg/kg compared with those treated with silymarin 100 mg/kg.

Considerable experimental and clinical evidence supports the prominent role of oxidative stress in the pathophysiological processes of liver injury related to CCl_4_ exposure (Yang et al., 2015; Zou et al., 2016). Lipid peroxidation is an autocatalytic mechanism that leads to the oxidative destruction of cellular membranes (Subudhi, Das, Paital, Bhanja, & Chainy, 2008; Videla, 2000). Such destruction can lead to cell death and to the production of toxic and reactive aldehyde metabolites called free radicals, of which MDA is one of the most important (Messarah et al., 2010; Venditti & Di Meo, 2006). It is known that reactive oxygen species (ROS) lead to the oxidative damage of biological macromolecules, including lipids, proteins, and DNA (Das & Chainy, 2001; Messarah et al., 2010), and that oxidative stress also influences adipocytes, causing decreases in body fat mass and, subsequently, body weight decrease (Voldstedlund, Tranum‐Jensen, Handberg, & Vinten, 1995). As MDA is a terminal product of lipid peroxidation, the content of MDA can be used to estimate the extent of lipid peroxidation (Messarah et al., 2010). Marked increases of liver MDA content have been observed in CCl_4_‐treated animals (Yang et al., 2015; Zou et al., 2016); in this study, MDA was increased at 24 h after single IP treatment of CCl_4_ 0.5 ml/kg. GSH is a representative endogenous antioxidant, which prevents tissue damage by suppression of ROS levels; furthermore, at certain cellular concentrations, it is accepted to be a protective antioxidant factor in tissues (Odabasoglu et al., 2006). SOD is another antioxidant enzyme that contributes to the enzymatic defense mechanisms and the enzyme CAT is responsible for the conversion of H_2_O_2_ to H_2_O (Cheeseman & Slater, 1993). Decreases in antioxidant enzyme activities, such as SOD and CAT, and decreases in the content of GSH may be indicative of the failure of cells to respond to the oxidative stress induced by CCl_4_ (Yang et al., 2015; Zou et al., 2016). Trichloromethyl radicals also react with the sulfhydryl groups of GSH leading to its deactivation (Al‐Sayed et al., 2014; Srivastava & Shivanandappa, 2010). In this experiment, the hepatic antioxidant defense system was clearly enhanced by treatment of all BH extracts; the effects of BHe were the strongest, followed by BHj and BHw, compared with the CCl_4_ control. Our results suggested that all BH extracts reduced the effect of CCl_4_‐induced liver injury through the augmentation of the hepatic antioxidant defense system. In particular, mice treated with BHe 200 mg/kg showed more favorable inhibitory effects on CCl_4_‐induced hepatic lipid peroxidation, with depletions of the endogenous antioxidant (GSH) and enzymes (SOD and CAT) observed, compared with silymarin 100 mg/kg‐treated mice, which supported the aforementioned hepatoprotective effects.

As previously reported (Ki et al., 2013; Lee et al., 2014), vacuolation (the deposition of lipid droplets), the ballooning of hepatocytes, and inflammatory cell infiltration were detected after single IP injection of CCl_4_ 0.5 ml/kg, which are indications of classic centrolobular necrosis. Damaged hepatocytes were mainly located around the central veins and the fatty changed cells were marginally located. CCl_4_ treatment‐related acute hepatic damages were confirmed by using HAI grading scores, based on the assessment of confluent necrosis, focal lytic necrosis, apoptosis, and focal and portal inflammation, which provides a well‐established semi‐quantitative histopathological scoring system in which higher grade scores indicate severe hepatitis (Ishak et al., 1995). The percentage of degenerative regions, the number of degenerative hepatocytes, and the numbers of inflammatory cells infiltrated in hepatic parenchyma were significantly increased in the CCl_4_ control compared with the intact control. However, the CCl_4_ treatment‐related centrolobular acute hepatic damage was significantly inhibited by the treatment of all BH extracts (in the order BHe, BHj, and BHw) compared with those of the CCl_4_ control. These histopathological findings were considered to provide direct and consistent evidence that all BH extracts tested in this study exerted favorable hepatoprotective effects on acute hepatic damage. In particular, mice treated with BHe 200 mg/kg showed more favorable hepatoprotective effects at the histopathological level on the CCl_4_‐induced centrolobular necrosis‐related acute liver damage compared with mice treated with silymarin 100 mg/kg.

Apoptosis occurs through two pathways, an extrinsic pathway that involves the interaction of death ligands with their respective cell surface receptors and an intrinsic pathway that is initiated by insults that damage the DNA, such as ultraviolet light and chemotherapeutic agents. Both pathways eventually result in mitochondrial damage, the release of cytochrome *c*, and the downstream activation of caspases, such as caspase‐3. The activation of other downstream caspases results in the cleavage of cellular proteins, such as PARP, cytokeratin 18, and other caspases, which lead to the morphologic and biochemical changes of apoptosis (Barrett, Willingham, Garvin, & Willingham, 2001; Nunez, Benedict, Hu, & Inohara, 1998). PARP is a nuclear DNA‐binding protein that functions in DNA base excision repair (Trucco, Oliver, de Murcia, & Menissier‐de Murcia, 1998). PARP cleavage results in decrease enzymatic repair functions and contributes to the progression of apoptosis, although it is not strictly necessary for apoptosis to proceed (Smulson et al., 1998). Caspase 3, a downstream effector caspase, is responsible for the cleavage of several critical nuclear targets in the apoptotic cascade, including the inhibitor of caspase‐activated deoxynuclease, which results in nuclear fragmentation, and PARP, which results in a defective DNA repair function (Smyth, Berman, & Bursztajn, 2002). It has been reported that severe apoptosis of liver hepatocytes has been observed in CCl_4_‐induced acute liver injury (Sun et al., 2011), and the inhibition of cleaved caspase‐3 and PARP have been regarded as hepatoprotective indicators (Jiang et al., 2012; Talwar et al., 2013; Yu et al., 2014). In our study, increased cleaved caspase‐3 and PARP immunoreactive hepatocytes were also demonstrated in the CCl_4_ control, mainly in the centrolobular regions, compared with the intact control. Noticeably, all BH extracts significantly reduced the CCl_4_‐induced increases in the number of cleaved caspase‐3 and PARP immunolabeled hepatocytes; at 200 mg/kg, BHe exerted the strongest effects, followed by BHj and BHw. These immunohistochemical results on the hepatic cleaved caspase‐3 and PARP immunostained cells provided direct evidence that the hepatoprotective effects on CCl_4_‐induced acute hepatic damage exerted by all BH extracts tested in this study may occur through anti‐apoptotic activity. BHe exerted the strongest effect, followed by BHj and BHw; in particularly, mice treated with BHe 200 mg/kg consistently demonstrated more favorable anti‐apoptotic effects against CCl_4_ treatment compared with those induced by silymarin.

NT is a product of tyrosine nitration mediated by reactive nitrogen species, such as the peroxynitrite anion and nitrogen dioxide. It is detectable in many pathological conditions, including CCl_4_‐induced acute and acute hepatic damages, and is considered to be marker of NO‐dependent, reactive nitrogen species‐induced nitrative stress (Lee et al., 2014; Pacher et al., 2007). 4‐HNE is an α, β‐unsaturated hydroxyalkenal produced by lipid peroxidation in cells; both these compounds are considered as possible causative agents of numerous diseases, including chronic inflammation, neurodegenerative diseases, adult respiratory distress syndrome, atherogenesis, diabetes, and different types of cancer (Lee et al., 2014; Smathers et al., 2011). The metabolism of CCl_4_ also initiates the peroxidation of polyunsaturated fatty acids that produce α, β‐unsaturated aldehydes, including 4‐HNE and malondialdehyde (Hartley, Kolaja, Reichard, & Petersen, 1999; Sigala et al., 2006). In the present study, marked and significant increases of NT and 4‐HNE immunostained hepatocytes were observed in the CCl_4_ control compared with the intact control, but were significantly reduced by the 7 day continuous pretreatment of all BH extracts at 200 mg/kg and by silymarin 100 mg/kg. In particular, mice treated with BHe 200 mg/kg showed better inhibitory effects on the CCl_4_‐induced increases of NT and 4‐HNE immunopositive hepatocytes in mice compared with silymarin 100 mg/kg‐treated mice. These immunohistochemical findings on the hepatic NT and 4‐HNE immunostained cells provided direct evidence that the hepatoprotective effects on the CCl_4_‐induced acute hepatic damages of all three different BH extracts detected in this study may occur through antioxidant effects.

## 5 CONCLUSION

Through the assessment of the key parameters of the hepatoprotective effects on CCl_4_‐induced acute liver injury in mice, the present work demonstrated that the oral pre‐administration of BHe, BHw, and BHj exerted favorable hepatoprotective effects through the activation of hepatic antioxidant defense systems; the strongest effects occurred in BHe, followed by BHw and BHj. In particular, BHe 200 mg/kg showed more favorable hepatoprotective effects compared with those of silymarin 100 mg/kg on CCl_4_‐induced acute liver damage in mice in the current study. Therefore, BHe is a suitable candidate BH extract for the development of potent hepatoprotective medicinal foods.

## CONFLICT OF INTEREST

The authors declare that there are no conflicts of interest.

## ETHICAL APPROVAL

This experiment was conducted in accordance with the international regulations of the usage and welfare of laboratory animals, and approved by the Institutional Animal Care and Use Committee of Daegu Haany University (Gyeongsan, Korea) (Approval No. DHU2016‐090).

## References

[fsn3893-bib-0001] Aebi, H. (1974). Catalase In Bergmeyer H. U. (Ed.), Methods in enzymatic analysis (pp. 673–686). New York, NY: Academic Press 10.1016/B978-0-12-091302-2.50032-3

[fsn3893-bib-0003] Al‐Sayed, E. , Abdel‐Daim, M. M. , Kilany, O. E. , Karonen, M. , & Sinkkonen, J. (2015). Protective role of polyphenols from Bauhinia hookeri against carbon tetrachloride‐induced hepato‐ and nephrotoxicity in mice. Renal Failure, 37, 1198–1207. 10.3109/0886022X.2015.1061886 26382171

[fsn3893-bib-0004] Al‐Sayed, E. , Martiskainen, O. , el‐Din, S. H. S. , Sabra, A. N. A. , Hammam, O. A. , & Abdel‐Daim, M. M. (2014). Hepatoprotective and antioxidant effect of *Bauhinia hookeri* extract against carbon tetrachloride‐induced hepatotoxicity in mice and characterization of its bioactive compounds by HPLC‐PDA‐ESI‐MS/MS. BioMed Research International, 2014, 245171. 24955350 10.1155/2014/245171 PMC4053259

[fsn3893-bib-0005] Barrett, K. L. , Willingham, J. M. , Garvin, J. A. , & Willingham, M. C. (2001). Advances in cytochemical methods for detection of apoptosis. Journal of Histochemistry and Cytochemistry, 49, 821–832. 10.1177/002215540104900703 11410607

[fsn3893-bib-0006] Berasain, C. , Castillo, J. , Perugorria, M. J. , Latasa, M. U. , Prieto, J. , & Avila, M. A. (2009). Inflammation and liver cancer: New molecular links. Annals of the New York Academy of Sciences, 1155, 206–221. 10.1111/j.1749-6632.2009.03704.x 19250206

[fsn3893-bib-0007] Boll, M. , Weber, L. W. , Becker, E. , & Stampfl, A. (2001). Mechanism of carbon tetrachloride‐induced hepatotoxicity. Hepatocellular damage by reactive carbon tetrachloride metabolites. Zeitschrift für Naturforschung C, 56(7–8), 649–659. 10.1515/znc-2001-7-826 11531102

[fsn3893-bib-0008] Chaovanalikit, A. , Thompson, M. M. , & Wrolstad, R. E. (2004). Characterization and quantification of anthocyanins and polyphenolics in blue honeysuckle (*Lonicera caerulea* L.). Journal of Agricultural and Food Chemistry, 52, 848–852. 10.1021/jf030509o 14969540

[fsn3893-bib-0009] Cheeseman, K. H. , & Slater, T. F. (1993). An introduction to free radical biochemistry. British Medical Bulletin, 49, 481–493. 10.1093/oxfordjournals.bmb.a072625 8221017

[fsn3893-bib-0010] Chen, L. , Xin, X. , Yuan, Q. , Su, D. , & Liu, W. (2014). Phytochemical properties and antioxidant capacities of various colored berries. Journal of the Science of Food and Agriculture, 94, 180–188. 10.1002/jsfa.6216 23653223

[fsn3893-bib-0011] Cheng, N. , Ren, N. , Gao, H. , Lei, X. , Zheng, J. , & Cao, W. (2013). Antioxidant and hepatoprotective effects of *Schisandra chinensis* pollen extract on CCl_4_‐induced acute liver damage in mice. Food and Chemical Toxicology, 55, 234–240. 10.1016/j.fct.2012.11.022 23201450

[fsn3893-bib-0012] Cordero‐Pérez, P. , Torres‐González, L. , Aguirre‐Garza, M. , Camara‐Lemarroy, C. , Guzmán‐de la Garza, F. , Alarcón‐Galván, G. , … Muñoz‐Espinosa, L. E. (2013). Hepatoprotective effect of commercial herbal extracts on carbon tetrachloride‐induced liver damage in Wistar rats. Pharmacognosy Research, 5, 150–156. 10.4103/0974-8490.112417 23900881 PMC3719254

[fsn3893-bib-0013] Cui, Y. , Yang, X. , Lu, X. , Chen, J. , & Zhao, Y. (2014). Protective effects of polyphenols‐enriched extract from Huangshan Maofeng green tea against CCl_4_‐induced liver injury in mice. Chemico‐Biological Interactions, 220(C), 75–83. 10.1016/j.cbi.2014.06.018 24973642

[fsn3893-bib-0014] Das, K. , & Chainy, G. B. (2001). Modulation of rat liver mitochondrial antioxidant defence system by thyroid hormone. Biochimica et Biophysica Acta, 1537, 1–13. 11476958 10.1016/s0925-4439(01)00048-5

[fsn3893-bib-0015] Ebaid, H. , Bashandy, S. A. , Alhazza, I. M. , Rady, A. , & El‐Shehry, S. (2013). Folic acid and melatonin ameliorate carbon tetrachloride‐induced hepatic injury, oxidative stress and inflammation in rats. Nutrition and Metabolism, 10, Article No. 20 10.1186/1743-7075-10-20 23374533 PMC3570377

[fsn3893-bib-0016] Fahmy, N. M. , Al‐Sayed, E. , Abdel‐Daim, M. M. , Karonen, M. , & Singab, A. N. (2016). Protective effect of *Terminalia muelleri* against carbon tetrachloride‐induced hepato and nephro‐toxicity in mice and characterization of its bioactive constituents. Pharmaceutical Biology, 54, 303–313. 10.3109/13880209.2015.1035794 25894213

[fsn3893-bib-0017] Ferreira, E. A. , Gris, E. F. , Felipe, K. B. , Correia, J. F. , Cargnin‐Ferreira, E. , Wilhelm Filho, D. , & Pedrosa, R. C. (2010). Potent hepatoprotective effect in CCl_4_‐induced hepatic injury in mice of phloroacetophenone from *Myrcia multiflora* . Libyan Journal of Medicine, 5, Article No. 4891 10.3402/ljm.v5i0.4891 PMC3071176 21483585

[fsn3893-bib-0018] Fox, J. G. , Cohen, B. J. , & Loew, F. M. (1984). Laboratory animal medicine. Orlando, FL: Academic Press Inc..

[fsn3893-bib-0019] Han, B. , Gao, Y. , Wang, Y. , Wang, L. , Shang, Z. , Wang, S. , & Pei, J. (2016). Protective effect of a polysaccharide from Rhizoma Atractylodis Macrocephalae on acute liver injury in mice. International Journal of Biological Macromolecules, 87, 85–91. 10.1016/j.ijbiomac.2016.01.086 26820352

[fsn3893-bib-0020] Hartley, D. P. , Kolaja, K. L. , Reichard, J. , & Petersen, D. R. (1999). 4‐Hydroxynonenal and malondialdehyde hepatic protein adducts in rats treated with carbon tetrachloride: Immunochemical detection and lobular localization. Toxicology and Applied Pharmacology, 161, 23–33. 10.1006/taap.1999.8788 10558920

[fsn3893-bib-0021] Ishak, K. , Baptista, A. , Bianchi, L. , Callea, F. , De Groote, J. , Gudat, F. , … Thaler, H. (1995). Histological grading and staging of chronic hepatitis. Journal of Hepatology, 22, 696–699. 10.1016/0168-8278(95)80226-6 7560864

[fsn3893-bib-0022] Jain, N. K. , Lodhi, S. , Jain, A. , Nahata, A. , & Singhai, A. K. (2011). Effects of *Phyllanthus acidus* (L.) Skeels fruit on carbon tetrachloride‐induced acute oxidative damage in livers of rats and mice. Zhong Xi Yi Jie He Xue Bao, 9, 49–56. 10.3736/jcim 21227033

[fsn3893-bib-0023] Jamall, I. S. , & Smith, J. C. (1985). Effects of cadmium on glutathione peroxidase, superoxidase dismutase and lipid peroxidation in the rat heart: A possible mechanism of cadmium cardiotoxicity. Toxicology and Applied Pharmacology, 80, 33–42. 10.1016/0041-008X(85)90098-5 4024106

[fsn3893-bib-0024] Jiang, W. , Gao, M. , Sun, S. , Bi, A. , Xin, Y. , Han, X. , … Luo, L. (2012). Protective effect of L‐theanine on carbon tetrachloride‐induced acute liver injury in mice. Biochemical and Biophysical Research Communications, 422, 344–350. 10.1016/j.bbrc.2012.05.022 22583898

[fsn3893-bib-0025] Jin, X. H. , Ohgami, K. , Shiratori, K. , Suzuki, Y. , Koyama, Y. , Yoshida, K. , … Ohno, S. (2006). Effects of blue honeysuckle (*Lonicera caerulea* L.) extract on lipopolysaccharide‐induced inflammation in vitro and in vivo. Experimental Eye Research, 82, 860–867. 10.1016/j.exer.2005.10.024 16309673

[fsn3893-bib-0026] Jurgoński, A. , Juśkiewicz, J. , & Zduńczyk, Z. (2013). An anthocyanin‐rich extract from Kamchatka honeysuckle increases enzymatic activity within the gut and ameliorates abnormal lipid and glucose metabolism in rats. Nutrition, 29, 898–902. 10.1016/j.nut.2012.11.006 23422536

[fsn3893-bib-0027] Kang, S. J. , Lee, J. E. , Lee, E. K. , Jung, D. H. , Song, C. H. , Park, S. J. , … Lee, Y. J. (2014). Fermentation with Aquilariae Lignum enhances the anti‐diabetic activity of green tea in type II diabetic db/db mouse. Nutrients, 6, 3536–3571. 10.3390/nu6093536 25207824 PMC4179175

[fsn3893-bib-0028] Kavutcu, M. , Canbolat, O. , Oztürk, S. , Olcay, E. , Ulutepe, S. , Ekinci, C. , … Durak, I. (1996). Reduced enzymatic antioxidant defense mechanism in kidney tissues from gentamicin‐treated guinea pigs: Effects of vitamins E and C. Nephron, 72, 269–274. 10.1159/000188853 8684538

[fsn3893-bib-0029] Ki, S. H. , Yang, J. H. , Ku, S. K. , Kim, S. C. , Kim, Y. W. , & Cho, I. J. (2013). Red ginseng extract protects against carbon tetrachloride‐induced liver fibrosis. Journal of Ginseng Research, 37, 45–53. 10.5142/jgr.2013.37.45 23717156 PMC3659625

[fsn3893-bib-0030] Lee, J. H. , Jang, E. J. , Seo, H. L. , Ku, S. K. , Lee, J. R. , Shin, S. S. , … Kim, Y. W. (2014). Sauchinone attenuates liver fibrosis and hepatic stellate cell activation through TGF‐β/Smad signaling pathway. Chemico‐Biological Interactions, 224C, 58–67. 10.1016/j.cbi.2014.10.005 25451574

[fsn3893-bib-0031] Levene, A. (1981). Pathological factors influencing excision of tumours in the head and neck. Part I”. Clinical Otolaryngology and Allied Sciences, 6, 145–151. 10.1111/j.1365-2273.1981.tb01800.x 7016380

[fsn3893-bib-0032] Lowry, O. H. , Rosenbrough, N. J. , Farr, A. L. , & Randall, R. J. (1951). Protein measurement with the Folin phenol reagent. Journal of Biological Chemistry, 193, 265–275. 14907713

[fsn3893-bib-0033] Lu, Y. , Hu, D. , Ma, S. , Zhao, X. , Wang, S. , Wei, G. , … Wang, J. (2016). Protective effect of wedelolactone against CCl_4_‐induced acute liver injury in mice. International Immunopharmacology, 34, 44–52. 10.1016/j.intimp.2016.02.003 26921731

[fsn3893-bib-0034] Ludbrook, J. (1997). Update: Microcomputer statistics packages. A personal view. Clinical and Experimental Pharmacology and Physiology, 24, 294–296. 10.1111/j.1440-1681.1997.tb01823.x 9131301

[fsn3893-bib-0035] Messarah, M. , Boumendjel, A. , Chouabia, A. , Klibet, F. , Abdennour, C. , Boulakoud, M. S. , & Feki, A. E. (2010). Influence of thyroid dysfunction on liver lipid peroxidation and antioxidant status in experimental rats. Experimental and Toxicologic Pathology, 62, 301–310. 10.1016/j.etp.2009.04.009 19540741

[fsn3893-bib-0036] Nunez, G. , Benedict, M. A. , Hu, Y. , & Inohara, N. (1998). Caspases: The proteases of the apoptotic pathway. Oncogene, 17, 3237–3245. 10.1038/sj.onc.1202581 9916986

[fsn3893-bib-0037] Odabasoglu, F. , Cakir, A. , Suleyman, H. , Aslan, A. , Bayir, Y. , Halici, M. , & Kazaz, C. (2006). Gastroprotective and antioxidant effects of usnic acid on indomethacin‐induced gastric ulcer in rats. Journal of Ethnopharmacology, 103, 59–65. 10.1016/j.jep.2005.06.043 16169175

[fsn3893-bib-0038] Pacher, P. , Beckman, J. S. , & Liaudet, L. (2007). Nitric oxide and peroxynitrite in health and disease. Physiological Reviews, 87, 315–424. 10.1152/physrev.00029.2006 17237348 PMC2248324

[fsn3893-bib-0039] Palíková, I. , Valentová, K. , Oborná, I. , & Ulrichová, J. (2009). Protectivity of blue honeysuckle extract against oxidative human endothelial cells and rat hepatocyte damage. Journal of Agricultural and Food Chemistry, 57, 6584–6589. 10.1021/jf9003994 19572653

[fsn3893-bib-0040] Park, S. I. , Lee, Y. J. , Choi, S. H. , Park, S. J. , Song, C. H. , & Ku, S. K. (2016). Therapeutic effects of blue honeysuckle on lesions of hyperthyroidism in rats. American Journal of Chinese Medicine, 44, 1441–1456. 10.1142/S0192415X16500804 27785940

[fsn3893-bib-0041] Pinto, C. , Duque, A. L. , Rodríguez‐Galdón, B. , Cestero, J. J. , & Macías, P. (2012). Xanthohumol prevents carbon tetrachloride‐induced acute liver injury in rats. Food and Chemical Toxicology, 50, 3405–3412. 10.1016/j.fct.2012.07.035 22884764

[fsn3893-bib-0042] Pradeep, K. , Mohan, C. V. , Anand, K. G. , & Karthikeyan, S. (2005). Effect of pretreatment of *Cassia fistula* Linn. leaf extract against subacute CCl_4_ induced hepatotoxicity in rats. Indian Journal of Experimental Biology, 43, 526–530. 15991578

[fsn3893-bib-0043] Sedlak, J. , & Lindsay, R. H. (1968). Estimation of total, protein‐bound, and nonprotein sulfhydryl groups in tissue with Ellman's reagent. Analytical Biochemistry, 25, 192–205. 10.1016/0003-2697(68)90092-4 4973948

[fsn3893-bib-0044] Sigala, F. , Theocharis, S. , Sigalas, K. , Markantonis‐Kyroudis, S. , Papalabros, E. , Triantafyllou, A. , … Andreadou, I. (2006). Therapeutic value of melatonin in an experimental model of liver injury and regeneration. Journal of Pineal Research, 40, 270–279. 10.1111/j.1600-079X.2005.00310.x 16499564

[fsn3893-bib-0045] Smathers, R. L. , Galligan, J. J. , Stewart, B. J. , & Petersen, D. R. (2011). Overview of lipid peroxidation products and hepatic protein modification in alcoholic liver disease. Chemico‐Biological Interactions, 192C, 107–112. 10.1016/j.cbi.2011.02.021 PMC3109208 21354120

[fsn3893-bib-0046] Smulson, M. E. , Pang, D. , Jung, M. , Dimtchev, A. , Chasovskikh, S. , Spoonde, A. , … Dritschilo, A. (1998). Irreversible binding of poly‐(ADP) ribose polymerase cleavage product to DNA ends revealed by atomic force microscopy: Possible role in apoptosis. Cancer Research, 58, 3495–3498. 9721847

[fsn3893-bib-0047] Smyth, P. G. , Berman, S. A. , & Bursztajn, S. (2002). Markers of apoptosis: Methods for elucidating the mechanism of apoptotic cell death from the nervous system. BioTechniques, 32, 648–665. 10.2144/02323dd02 11911667

[fsn3893-bib-0048] Sodikoff, C. H. (1995). Laboratory profiles of small animal diseases: A guide to laboratory diagnosis (pp. 1–36). St. Louise, MO: Mosby.

[fsn3893-bib-0049] Srivastava, A. , & Shivanandappa, T. (2010). Hepatoprotective effect of the root extract of *Decalepis hamiltonii* against carbon tetrachloride‐induced oxidative stress in rats. Food Chemistry, 118, 411–417. 10.1016/j.foodchem.2009.05.014

[fsn3893-bib-0050] Subudhi, U. , Das, K. , Paital, B. , Bhanja, S. , & Chainy, G. B. (2008). Alleviation of enhanced oxidative stress and oxygen consumption of L‐thyroxine induced hyperthyroid rat liver mitochondria by vitamin E and curcumin. Chemico‐Biological Interactions, 173, 105–114. 10.1016/j.cbi.2008.02.005 18377885

[fsn3893-bib-0051] Sun, H. , Chen, L. , Zhou, W. , Hu, L. , Li, L. , Tu, Q. , … Wang, H. (2011). The protective role of hydrogen‐rich saline in experimental liver injury in mice. Journal of Hepatology, 54, 471–480. 10.1016/j.jhep.2010.08.011 21145612

[fsn3893-bib-0052] Sun, Y. , Larry, W. O. , & Ying, L. (1988). A simple method for clinical assay of superoxide dismutase. Clinical Chemistry, 34, 497–500. 3349599

[fsn3893-bib-0053] Svarcova, I. , Heinrich, J. , & Valentova, K. (2007). Berry fruits as a source of biologically active compounds: The case of *Lonicera caerulea* . Biomedical Papers of the Medical Faculty of the University Palacky, Olomouc, Czechoslovakia, 151, 163–174. 10.5507/bp.2007.031 18345248

[fsn3893-bib-0054] Svobodová, A. , Rambousková, J. , Walterová, D. , & Vostálová, J. (2008). Protective effects of phenolic fraction of blue honeysuckle fruits against UV A‐induced damage to human keratinocytes. Archives of Dermatological Research, 300, 225–233. 10.1007/s00403-008-0850-5 18404271

[fsn3893-bib-0055] Tajima, Y. (1989). Biological reference data book on experimental animals. Tokyo, Japan: Soft Science Inc..

[fsn3893-bib-0056] Talwar, S. , Jagani, H. V. , Nayak, P. G. , Kumar, N. , Kishore, A. , Bansal, P. , … Nandakumar, K. (2013). Toxicological evaluation of *Terminalia paniculata* bark extract and its protective effect against CCl_4_‐induced liver injury in rodents. BMC Complementary and Alternative Medicine, 13, Article no. 127 10.1186/1472-6882-13-127 23742226 PMC3682919

[fsn3893-bib-0057] Trucco, C. , Oliver, F. J. , de Murcia, G. , & Menissier‐de Murcia, J. (1998). DNA repair defect in poly (ADP‐ribose) polymerase‐deficient cell lines. Nucleic Acids Research, 26, 2644–2649. 10.1093/nar/26.11.2644 9592149 PMC147627

[fsn3893-bib-0058] Vargas‐Mendoza, N. , Madrigal‐Santillán, E. , Morales‐González, A. , Esquivel‐Soto, J. , Esquivel‐Chirino, C. , García‐Luna, Y. , … Morales‐González, J. A. (2014). Hepatoprotective effect of silymarin. World Journal of Hepatology, 6, 144–149. 10.4254/wjh.v6.i3.144 24672644 PMC3959115

[fsn3893-bib-0059] Venditti, P. , & Di Meo, S. (2006). Thyroid hormone‐induced oxidative stress. Cellular and Molecular Life Sciences, 63, 414–434. 10.1007/s00018-005-5457-9 16389448 PMC11136030

[fsn3893-bib-0060] Videla, L. A. (2000). Energy metabolism, thyroid calorigenesis, and oxidative stress: Functional and cytotoxic consequences. Redox Report, 5, 265–275. 10.1179/135100000101535807 11145101

[fsn3893-bib-0061] Voldstedlund, M. , Tranum‐Jensen, J. , Handberg, A. , & Vinten, J. (1995). Quantity of Na/K‐ATPase and glucose transporters in the plasma membrane of rat adipocytes is reduced by in vivo triiodothyronine. European Journal of Endocrinology, 133, 626–634. 10.1530/eje.0.1330626 7581995

[fsn3893-bib-0062] Vostálová, J. , Galandáková, A. , Palíková, I. , Ulrichová, J. , Doležal, D. , Lichnovská, R. , … Rajnochová Svobodová, A. (2013). *Lonicera caerulea* fruits reduce UVA‐induced damage in hairless mice. Journal of Photochemistry and Photobiology B, 128, 1–11. 10.1016/j.jphotobiol.2013.07.024 23974431

[fsn3893-bib-0063] Wang, R. , Feng, X. , Zhu, K. , Zhao, X. , & Suo, H. (2016). Preventive activity of banana peel polyphenols on CCl_4_‐induced experimental hepatic injury in Kunming mice. Experimental and Therapeutic Medicine, 11, 1947–1954. 10.3892/etm.2016.3155 27168833 PMC4840597

[fsn3893-bib-0064] Wang, D. H. , Wang, Y. N. , Ge, J. Y. , Liu, H. Y. , Zhang, H. J. , Qi, Y. , … Cui, X. L. (2013). Role of activin A in carbon tetrachloride‐induced acute liver injury. World Journal of Gastroenterology, 19, 3802–3809. 10.3748/wjg.v19.i24.3802 23840118 PMC3699031

[fsn3893-bib-0065] Weber, L. W. , Boll, M. , & Stampfl, A. (2003). Hepatotoxicity and mechanism of action of haloalkanes: Carbon tetrachloride as a toxicological model. Critical Reviews in Toxicology, 33, 105–136. 10.1080/713611034 12708612

[fsn3893-bib-0066] Wellington, K. , & Jarvis, B. (2001). Silymarin: A review of its clinical properties in the management of hepatic disorders. BioDrugs: Clinical Immunotherapeutics, Biopharmaceuticals and Gene Therapy, 15, 465–489. 10.2165/00063030-200115070-00005 11520257

[fsn3893-bib-0067] Yang, J. , Li, Y. , Wang, F. , & Wu, C. (2010). Hepatoprotective effects of apple polyphenols on CCl_4_‐induced acute liver damage in mice. Journal of Agricultural and Food Chemistry, 58, 6525–6531. 10.1021/jf903070a 20415417

[fsn3893-bib-0068] Yang, B. Y. , Zhang, X. Y. , Guan, S. W. , & Hua, Z. C. (2015). Protective effect of procyanidin B2 against CCl_4_‐induced acute liver injury in mice. Molecules, 20, 12250–12265. 10.3390/molecules200712250 26151119 PMC6332456

[fsn3893-bib-0069] Yu, H. , Zheng, L. , Yin, L. , Xu, L. , Qi, Y. , Han, X. , … Peng, J. (2014). Protective effects of the total saponins from *Dioscorea nipponica* Makino against carbon tetrachloride‐induced liver injury in mice through suppression of apoptosis and inflammation. International Immunopharmacology, 19, 233–244. 10.1016/j.intimp.2014.01.019 24491258

[fsn3893-bib-0070] Zdařilová, A. , Svobodova, A. R. , Chytilová, K. , Šimánek, V. , & Ulrichová, J. (2010). Polyphenolic fraction of *Lonicera caerulea* L. fruits reduces oxidative stress and inflammatory markers induced by lipopolysaccharide in gingival fibroblasts. Food and Chemical Toxicology, 48, 1555–1561. 20332009 10.1016/j.fct.2010.03.024

[fsn3893-bib-0071] Zhao, H. , Wang, Z. , Ma, F. , Yang, X. , Cheng, C. , & Yao, L. (2012). Protective effect of anthocyanin from L*onicera caerulea* var. *edulis* on radiation‐induced damage in mice. International Journal of Molecular Sciences, 13, 11773–11782. 10.3390/ijms130911773 23109882 PMC3472774

[fsn3893-bib-0072] Zou, J. , Qi, F. , Ye, L. , & Yao, S. (2016). Protective Role of grape seed proanthocyanidins against CCl_4_ induced acute liver injury in mice. Medical Science Monitor, 22, 880–889. 10.12659/MSM.895552 26986029 PMC4801141

